# Process strengths determine the forms of the relationship between plant species richness and primary productivity

**DOI:** 10.1371/journal.pone.0185884

**Published:** 2017-11-15

**Authors:** Zhenhong Wang

**Affiliations:** 1 School of Environmental Science and Engineering, Chang`an University, Xi`an, China; 2 Key Laboratory of Subsurface Hydrology and Ecological Effects in Arid Region, Ministry of Education, Chang`an University, Xi`an, China; University of Waikato, NEW ZEALAND

## Abstract

The current rates of biodiversity loss have exceeded the rates observed during the earth’s major extinction events, which spurs the studies of the ecological relationships between biodiversity and ecosystem functions, stability, and services to determine the consequences of biodiversity loss. Plant species richness-productivity relationship (SRPR) is crucial to the understanding of these relationships in plants. Most ecologists have reached a widespread consensus that the loss of plant diversity undoubtedly impairs ecosystem functions, and have proposed many processes to explain the SRPR. However, none of the available studies has satisfactorily described the forms and mechanisms clarifying the SRPR. Observed results of the SRPR forms are inconsistent, and studies have long debated the ecological processes explaining the SRPR. Here, I have developed a simple model that combines the positive and/or negative effects of sixteen ecological processes on the SRPR and models that describe the dynamics of complementary-selection effect, density effect, and the interspecific competitive stress influenced by other ecological processes. I can regulate the strengths of the effects of these ecological processes to derive the asymptotic, positive, humped, negative, and irregular forms of the SRPR, and verify these forms using the observed data. The results demonstrated that the different strengths of the ecological processes determine the forms of the SRPR. The forms of the SRPR can change with variations in the strengths of the ecological processes. The dynamic characteristics of the complementary-selection effect, density effect, and the interspecific competitive stress on the SRPR are diverse, and are dependent on the strengths and variation of the ecological processes. This report explains the diverse forms of the SRPR, clarifies the integrative effects of the different ecological processes on the SRPR, and deepens our understanding of the interactions that occur among these ecological processes.

## Introduction

Plant species richness and primary productivity are two fundamental properties of ecosystems [1, 2]. In recent decades, unprecedented global loss of plant diversity has led to an increasingly pressing need for the comprehensive understanding of whether this loss will greatly impair primary productivity and further ecosystem functioning and services [3,4]. Most ecologists hold that plant diversity directly promotes primary productivity and significantly enhances ecosystem functioning and services [5–7]. However, there is still a great debate on the shapes of the plant species richness-primary productivity relationship (SRPR) and its underlying mechanisms [8,9]. The mechanisms explaining the SRPR include many processes, such as complementarity effects, selection effects, density effects, and intermediate disturbance [5,6,10,11]. Specifically, the observed dominant form or pattern of the SRPR is asymptotic or positive, such as in the manipulated diversity-productivity studies and in natural grasslands [5,6]. However, the dominant form has been challenged by humped, negative monotonic, and even irregular patterns over the last several decades [12–23]. The explanatory power of these processes through some mechanisms has also been debated, such as competition stress, negative selection effect, or competitive exclusion having been suggested to affect the productivity, species richness, and interactions [12–23].

Strictly speaking, to date, these issues on the forms and mechanisms of the SRPR have not been completely solved. Several reviews have argued that the SRPR is complex because it is governed by various biotic and abiotic processes and different scales that affect species richness, productivity, and interactions, which increases the difficulty of determining a single general pattern [11,24,25]. However, the processes affecting the SRPR are so many that they could not all be tested simultaneously in empirical studies; thus, ecologists are motivated to use a theoretical combination of processes to test the comprehensive effects on the SRPR. For example, the resource-based models combine species coexistence and plant competition for a limiting soil nutrient to elucidate complementarity effects and positive correlations between the mean resource-use intensity and plant species richness [26,27]. The dynamic models combining various trophic interactions (e.g., grazing, predation and soil nutrients), and nontrophic interactions (e.g., light limitation and water stress)indicate that light competition and water stress among plants in communities are weakened because of plant diversity, and consequently biomass production increases [28,29]. Comparatively, the recently published models demonstrate how the strengths of complementarity and selection effects on biomass production are influenced by trait and environmental variability, resource distribution, and species pool size [7]. The niche efficiency in complementarity effects is also suggested to influence plant productivity [30].

However, these studies have primarily highlighted the positive effects of the combined processes on the SRPR, but the negative effects are rarely considered [1,2,11,24]. This clearly hinders understanding of the patterns of the SRPR and its underlying mechanisms. Moreover, the SRPR includes two relationships: that which defines species richness as the independent variable and primary productivity as the dependent variable, and that which presents an opposite definition of these variables compared with the first relationship. The first relationship emphasizes ecosystem functioning and the effects of species richness, or a consequence of the loss of species richness. The second relationship focuses on the patterns of plant diversity on different levels of primary productivity and underlying mechanisms explaining the effects of primary productivity on plant diversity [31,32]. One review holds that many studies have not clearly defined the independent and dependent variables, which has led to confusion [24].

Here, I combine the effects of key ecological processes that are widely accepted to affect the SRPR, through establishment of mathematical models. Then, the model parameters that represent the strengths of these processes are changed to derive and identify the forms of the SRPR, and the derived SRPR forms are verified by the data from the observed studies of third parties. In these models, species richness is explicitly defined as the independent variable affecting primary productivity, and primary productivity is defined as the dependent variable. I assume that ecological processes that have a positive and/or negative effect on the SRPR vary temporally or spatially with increasing species richness. The process having a strongly positive effect on the SRPR at one species richness level may have a weakly positive or negative effect at another richness level. If the respective effects of these processes on the SRPR are reliable and acceptable, then the combined predictions must simultaneously hold true. The comprehensive effects of these positive and/or negative processes will determine the patterns of the SRPR.

## Materials and methods

### Combined key processes

There are five combined key processes. These include selection and complementarity effects, density effects, inter-specific competitive stress, disturbances, and resource availability.

(I)Selection and complementarity (SC) effects are those in which the selection effect is the standard statistical covariance effect. Specifically, species yields in a plant community are on average higher than the weighted average monoculture yield of the component species because a diverse community stochastically contains highly productive species [1,31,33]. The complementarity effect actually refers to an effect caused by differentiation in resource use and/or facilitative interactions among plant species, which become the main drivers of increased productivity at higher levels of species richness [1,2,5]. The selection effect is challenged, however, by the so-called zero-sum game, which states that in a diverse community, the less productive species also occur at high probability and offset the effect of highly productive species, thereby reducing the effect to zero [2,27,33]. Thus, in this model, I considered the two effects as one integral SC effect.

(II)Density effects are those in which positive relationships between plant productivity and the total number of plants in plant communities are based on *species-energy theory*, an effect that appears to have been previously ignored [34–36]. In the relationship, the total number of plants is likewise dependent on plant species richness [35–37]. Consequently, when the total number of plant individuals increases with an increase of plant species richness, productivity of a plant community presents an increasing trend at low interspecific and intraspecific competition levels. However, interspecific and intraspecific competition stress occurs at high species richness levels to reduce the mass of individual plants, and an increased number of individuals may conversely lead to low plant productivity [36,38–40].

(III)Inter-specific competitive stress is an important process that may generate a decline in primary productivity and species richness, but primary productivity also increases with the inter-specific competitive exclusion, leading to a local extinction of some subordinate species in plant communities [36,40,41]. Most competition theories indicate that mortality is not equal among plant species and that competitive exclusion reduces plant species richness in habitats with abundant resources and high plant productivity [36]. Specifically, the dominant species in resource acquisition and growth suppress the subordinate species, eventually excluding them and thereby yielding a relatively species-poor assemblage, which can conversely weaken the inter-specific competitive stress [36,42,43].

(IV)Disturbances may be natural or anthropogenic, and include such things as grazing, fire, or severe windstorms. Disturbances reduce plant productivity and species richness through a negative mortality-causing effect, and further regulate the SRPR [44–46]. However, moderate intensity of grazing can often restrain the dominant plants in grassland to weak the exclusion of subordinate species [47]. Moderate intensity fires can burn off litter and secondary metabolites of plants, hampering the establishment of immigrated species in forests [47–49]. Consequently, the moderate disturbances of grazing and fires generate high plant diversity and productivity [47–49]. Thus, disturbance can have both negative and positive effects on productivity, species richness and the SRPR.

(V)Resource availability refers to the available supply of sunlight, heat, nutrients, and water for plant establishment and growth. Resource availability promotes primary productivity and species richness and has a positive effect on the SRPR [28,41,50]. Resource availability includes two characteristics, i.e., the summed abundance of various resource types and the abundance of limiting resources [41,51]. The latter ensures weaker competitors to be capable of capturing the limiting resources not being excluded based on resource ratio theory [41,51].

### Combination model

Primary productivity, *P*(s), is affected by SC effect, *u*(s), and density effect, *m*(s), according to the premise that species richness, *s*, gradually increases in plant communities, which increases the likelihood of highly productive species and various forms of niche partitioning [1,2]. These allow plants to capture resources using methods that are complementary in space or time, and the species richness may increase the number of plant individuals and favorable interspecific interactions [1–3,5]. Resource availability (*R*_a_) promotes increases in species richness and enhances potential selection effects, complementarity effects and density effects on primary productivity [47,51–54]; however, the inter-specific competitive stress and strong natural and human disturbances reduce primary productivity and species richness, and weaken the potential SC effects and density effects on primary productivity [43,44,55]. All of these positive and negative processes regulate *P*(s), which are directly or indirectly related to species richness [10,56]. When species richness presents continual spatial or temporal increases or decreases, the *u*(s) and *m*(s) on the *P*(s) also continually increase or decrease [57]. Thus, I developed a simple differential equation (Eq 1) to combine these processes and tested how these processes affected the SRPR forms:

$$\frac{dy}{dx}=u\left(s\right)P+m\left(s\right)P-\tau DP+\mu R_{a}P$$ (1)

Where *P*(s) represents primary productivity (per unit time, *kg*.*ha*^-1^.*y*^-1^) and *s* represents species richness. When *dP*/*d*s in Eq 1 is greater than, equal to, or less than 0, then *P*(s) increases, stabilizes or declines, respectively. The parameters *u*(s)and *m*(s) represent the SC effects (*P*.*s*^-1^) and density effects (*P*.*s*^-1^) of *s*. The *u*(s) primarily enhances the mass of individual plants to increase *P*(s), whereas *m*(s) reflects a characteristic of plant species richness, *s*, by increasing plant density in a community to increase primary productivity, which is similar to *k* and *r* selection in plant strategy. Therefore, *u*(s) and *m*(s)were considered independent effects on *P*(s), and *u*(s)×*P* and *m*(s)×*P* were used to reflect the contribution of *u*(s) and *m*(s) to *P*(s) with increasing *s*. *D* represents a disturbance (without unit) in Eq 1, which is an impulse function. When *s* ≠ *s*_D_, *δ* (*s*_D_) = *d*Δ(*s*_D_)/*d*s = 0, and the disturbance does not occur, where *s*_D_ is the species richness levels on which the stochastic disturbance occurs. When *s* = *s*_D_, *δ* (*s*_D_) = *d*Δ(*s*_D_)/*d*s = 1, then *D* occurs. Therefore, at any scale, a lack of *D* produces the term, *τDP* = 0.*τ* is a transfer coefficient (*P*.*D*^-1^). Resource availability (*R*_*a*_, without unit) has a positive effect on *P*(s), and different *R*_*a*_ values occur among different habitats within a metacommunity or in different biogeographical provinces. However, because *R*_*a*_ is basically identical or similar among plots in a local plant community, the levels of *R*_*a*_ can be considered zero. *μ* is a transfer coefficient (*P*.*R*_*a*_^-1^). Both *D* and *R*_a_ in Eq 1 are to some extent related to the *s*, which makes Eq 1 homogeneous[48,51].

$$u\left(s\right)=as-k_{1}\text{ln}N$$ (2)

Eq 2 was used to determine changes in *u*(s) with increasing species richness in Eq 1. At low species richness, increases in temporal or spatial species richness result in an increased likelihood of highly productive species (i.e., selection effects) and the co-occurrence of species through niche partitioning and facilitation (i.e., complementarity effects), which yield positive *u*(s) on the *P*(s), i.e., increase the *P*(s)[3,5]. Here, *as* was used to reflect increases in the positive effects of species richness on *P*(s) (Eq 2). The coefficient *a* (*P*.*s*^-2^) is the intensity of the positive effect when species richness is increased within a plant community. However, when species richness increases to a higher level, then the interspecific competitive stress (ln*N*≥0;the unit of which is defined as *s*) begins to increase. The ln*N* is gradually strengthened with increasing species richness because plant species that have occupation of similar niches continually join and compete for resources [40,41,58]. The gradually strengthened ln *N* weakens the increasing *as*[36,40]. Here, *k*_1_ln*N* represents the decrease of *u*(s) in Eq 2, and *k*_1_ represents a transfer coefficient(*P*.*s*^-2^). Thus, the effect of *u*(s)on *P*(s)in Eq 1 is dictated by the balance between *as* and *k*_1_ln*N*. In Eq 2, when *s* = 1, the interspecific competitive stress does not exist and the ln*N* = 0 and *N* = 1; when *s* = 0, Eq 2 has no meaning in practice.

$$m\left(s\right)=bs-k_{2}\text{ln}N$$ (3)

Similarly, absent or weak inter-specific competitive stress ln*N* (*s*) occurs in the plant community when species richness is very low, and the size and mass of individual plants of each plant species in the community are not influenced by inter-specific stress ln*N* [36,39]. Thus, the total individual number and mass of the plant community increases with increasing plant species richness at the low species richness, which leads to high biomass production, i.e., a positive density effect *m*(s) on *P*(s), which can be represented using *bs* (Eq 3). The coefficient *b*(*P*.*s*^-2^) is the intensity of the positive effect. Many studies have shown that the number and mass of the total individual plants increased as a power function or function similar to a power function with increasing species richness [34–37], which is equivalent to the primitive function for the term *bs* in Eq 3 (the primitive function of the *bs* is combined by substitution into the following Eqs 10 and 11 to describe the relationship between species richness and density). However, the average size and mass of individual plants declines at high diversity levels because of strengthened interspecific stress ln*N*, although increasing diversity results in a greater number of individual plants in the community, which decreases the *P*(s). This is the negative density effect *m*(s)of species richness on *P*(s), which is represented by *k*_2_ln*N*in Eq 3. *k*_2_ is the transfer coefficient (*P*.*s*^-2^). The effect of *m*(s)on *P*(s)in Eq 1 is dictated by the balance between *bs* and *k*_2_ln*N*.

Because the effect of the interspecific competitive stress on *P*(s)is hysteretic with increasing species richness temporally or spatially, I used a log-transformation (ln*N*) in Eqs 2 and 3 to represent it. Moreover, I used a differential equation with one order to describe the increases of *N*(s) along a gradient of species richness (Eq 4) [40]. The first term *b*_1_*s* in Eq 4 represents the contributions from both gradually increasing species richness and consequently increasing productivity to the *N*[28,33,40]. *b*_1_ is the coefficient of the effect of increases in species richness (*N*.*s*^-2^). The term *b*_2_*P*_*m*_ represents the role of primary productivity that is unrelated to species richness, and *b*_2_ is the coefficient of the effect of the primary productivity (*N*.*P*^-2^). *P*_*m*_ is the (average) primary productivity of a monoculture of component species in the plant community (*kg*.*ha*^-1^.*y*^-1^). *D* may suppress the dominant species and consequently reduce *N*(s) among plant species, besides directly decreasing the *P*(s) and *s* in Eq 1. However, with increasing species richness, the plant community has an increasing resistance to *D*, which weakens the negative effect of *D* on *N*(s) [47–49]. Thus, -*ρD*/*s* was used to represent a contribution of *D* to *N*(s) (Eq 4) and *ρ* is a transfer coefficient (*Ns*.*D*^-2^).

$$\frac{\text{dN}}{\text{ds}}=({\text{b}}_{1}\text{s}+{\text{b}}_{2}{\text{P}}_{\text{m}}-\frac{\rho \text{D}}{\text{s}})\text{N}$$ (4)

*N* in Eq 4 can be directly integrated with the integration constant, which is zero:

$$\text{ln}N=0.5b_{1}{\text{s}}^{2}+b_{2}P_{m}s-\rho D\;\text{ln}\;\left(s\right)$$ (5)

To substitute Eq 5 into Eqs 2 and 3, the *u*(s) and *m*(s) of species richness can be written as follows:

$$u\;\left(\text{s}\right)=as-k_{1}\left(0.5b_{1}s^{2}+b_{2}P_{m}s-\rho D\;\text{ln}\left(s\right)\right)$$ (6)

$$m\left(s\right)=bs-k_{2}\left(0.5b_{1}s^{2}+b_{2}P_{m}s-\rho D\text{ln}\left(s\right)\right)$$ (7)

After Eqs 6 and 7 have been substituted into Eq 1, Eq 1 becomes the following:

$$\frac{dP}{ds}=\left[\left(a+b\right)s-\left(k1+k2\right)\left(0.5b_{1}s^{2}+b_{2}P_{m}s-\rho D\;\text{ln}\left(s\right)\right)\right]P-\tau DP+\mu R_{a}P$$ (8)

The variables in Eq 8 can be separated and *P*(s) can be integrated as follows:

$$\text{ln}\;P=C+\frac{1}{2}\left(a+b\right)s^{2}-\left(k_{1}+k_{2}\right)\left[\frac{1}{6}b_{1}s^{3}+\frac{1}{2}b_{2}P_{m}s^{2}-\rho D\left(s\;\text{ln}\left(\text{s}\right)-s\right)\right]-\tau Ds+\mu R_{a}s$$ (9)

where *C* is an integration constant. When *s* = 0, ln*P* = *P*_0_ = *P*_m_ = 0; then, *C* = 0, and Eq 9 changes as follows:

$$\text{ln}P=\frac{1}{2}\left(a+b\right)s^{2}-\left(k_{1}+k_{2}\right)\left[\frac{1}{6}b_{1}s^{3}+\frac{1}{2}b_{2}P_{m}s^{2}-\rho D\left(s\;\text{ln}\left(\text{s}\right)-s\right)\right]-\tau Ds+\mu R_{a}$$ (10)

When *s* = 1, ln*P* = *P*_1_ = *P*_*m*_ln, then $\text{C}={\text{P}}_{\text{m}}-\frac{1}{2}\left(a+b\right)+\left(k_{1}+k_{2}\right)\left(\frac{1}{6}b_{1}+\frac{1}{2}b_{2}P_{m}+1\right)+\tau \text{D}-{\mu \text{R}}_{\text{a}}$*C*, Eq 9 changes as follows:

$$\text{lnP}={\text{P}}_{\text{m}}+\frac{1}{2}\left(\text{a}+\text{b}\right)\left({\text{s}}^{2}-1\right)+\left(k_{1}+k_{2}\right)\left[\frac{1}{6}b_{1}(1-s^{3}\right)+\frac{1}{2}b_{2}P_{m}(1-s^{2})+\rho Ds\;\text{ln}\left(\text{s}\right)-\rho Ds+1]-\tau D\left(s-1\right)+\mu R_{a}\left(s-1\right)$$ (11)

Eq 11 is a final integration model combining the primary processes affecting SRPR. The parameters of all of these processes and their specific ecological significance in Eqs 1–11 are indicated in Table 1. When *as*>*k*_*1*_*N* and *bs*>*k*_*2*_*N* in Eqs 2 and 3, i.e., *u*(*s*) and *m*(*s*) >0, and there are no or only weak disturbances, then *dP*/*ds* in Eq 1 is positive and ln*P* in Eq 11 increases. When *as*<*k*_*1*_*N* and *bs*< *k*_*2*_*N*, i.e., *u*(*s*) and *m*(*s*) <0, and the product of *μR*_*a*_ is verysmall, then *dP*/*ds* in Eq 1 is negative and ln *P* decreases. When *as* = *k*_1_*N* and *bs* = *k*_2_*N*, i.e., *u*(*s*) and *m*(*s*) equal zero, and *μR*_*a*_ = *τD* in Eq 1, then *dP*/*ds* = 0 and ln*P* remains stable and does not increase or decrease. Especially when *R*_a_ = 0 in Eqs 1–11, these models may describe the SRPR and the dynamics of the *u*(s), *m*(s) and ln*N* in local natural or species-manipulated plant communities. When *R*_a_ ≠ 0 in Eqs 1–11, these models can be used to describe the SRPR and the dynamics of the *u*(s), *m*(s), and ln *N* across different local plant communities within a metacommunity or across local communities in different biogeographical provinces.

**Table 1:** The parameters and variables in Eqs 1–11.

Symbol	Ecological processes	The positive (+) and negative (-)effects of ecological processes on productivity, and citations	The assigned parameter values				
			Asymptotic form^#^	Positive form^#^	Humped form^#^	Negative form^#^	Irregular form^#^
D*	Disturbance intensity	(- or +) [44,45,47,48]	0,50, 2–50	0,0, 50	0,30, 50	0,0,60	0~100,0,17
τ*	Effect coefficients of disturbance on productivity(*P*.*D*^-1^)	(-or +)[44,45,47,48]	0,0,0.07	0,0.5, 0.02	0,0.2, 0.02	0,0.5,0.5	0.5,0.5,0.5
*R*_*a*_	Resource availability	(+)[28,49,52]	0,0.5,27–75	0,0,0.5	0,15,0.49	0,0,50	0,0,15–18
*μ*	Effect coefficient of resource availability on productivity (*P*.*R*_*a*_^-1^)	(+) [49,51]	0,0.005,0.46	0,0.5,0.001	00.137,0.001	0,0.5,0.5	0,0,0.5
*a*	Effect coefficients of species richness on SC effects (*P*.*s*^-2^)	(+)[2,3,5]	0.13,0.24,0.45	0.35,1.4,5.5	0.11,1.5, 0.24	0.08,0.13,0.13	0.03,1.5,0.2
*k*_*1*_	Effect coefficient of interspecific competitive stress on SC effects(*P*.*s*^-2^)	(-)[30–43]	0.06,0.07,0.02	0.04,0.0001,0.08	0.07,0.13, 0.13	0.09,0.14,0.14	0.07,0.0002, 0.15
*b*	Effect coefficient of species richness on density effects (*P*.*s*^-2^)	(+)[9,34–36,65]	0.15,0.23,0.55	0.25,1.2, 6.5	0.165,1.8, 0.13	0.04,0.15,0.15	0.07,2,0.28
*k*_*2*_	Effect coefficient of interspecific competitive stress on density effects (*P*.*s*^-2^)	(-)[9,35,36,44,65]	0.09.0.08,0.06	0.07,0.0002,0.07	0.1,0.17, 0.18	0.12,0.02,0.02	0.05,0.0005,0.25
*P*_*m*_	Primary productivity of a monoculture (kg.ha^-1^.y^-1^)	(-) [1,2]	40,204,518	0,620, 1354	100,950, 2	600,850, 135	150,547,350
*ρ*	Effect coefficient of disturbance on interspecific competitive stress(*Ns*.*D*^*-2*^)	(+)[44,45,47,48]	0.03,0.035,0.35	0.03,0.03,0.002	0.03,0.12, 0.002	0.03,0.03, 0.01	0.03,0.3,0.5
*b*_*1*_	Effect coefficient of species richness on interspecific competitive stress(*N*.*s*^*-2*^)	(-)[33,41–43]	0.090.11,0.002	0.04,0.0002, 0.001	0.11,0.8, 0.111	0.12,0.15, 0.04	0.11,0.00002 0.04
*b*_*2*_	Effect coefficient of primary productivity in monoculture on *N*(*N*.*P*^*-2*^)	(-)[40,41,42,43]	0.0005,0.0012,0.001	0.0007,0.0007,0.012	0.001,0.0006, 0.001	0.003,0.2,0.002	0.001,0.00001,0.023
*N*	Interspecific competitive stress	(-)[33,41–43]	Dependent variable or independent variable				
*u*(*s*)	SC effects(*P*.*s*^-1^)	(+) [2,3,5]	Dependent variable				
*m*(*s*)	Density effects(*P*.*s*^-1^)	(+) [9, 34–36]	Dependent variable				
s*	Species richness (*s*)	(+or-) [2,3,5]	Presenting a gradients along the x-axis				
*P*	Primary productivity (*kg*.*ha*^-1^.*y*^-1^)	(-) [1,2]	Dependent variable				

*represents the processes that have several effects, but these effects are separated in models. For example, species richness has a positive effect on productivity, but high species richness will lead to an intense interspecific competition stress causing the decreases in productivity. The first value in each cell of the data columns with #for derivation of the five typical forms of the SRPR was estimated using the stochastic approximation method [61]. The second value in each cell of the data columns with #was estimated using both the least-square method and the stochastic approximation method based on the observed productivity and species richness at local sites in experimental grasslands of Europe [62], the floodplain of the river Saale near Jena in Germany [63], the grasslands in Texas [64], natural plant communities in Gloucestershire of the UK [15] and the Czech Republic [22], respectively (Text B in S1 File). The estimated values may to some degree reflect the strengths of the processes affecting the SRPR in these sites. The third value in each cell of the data columns with # was also estimated using both the least-square method and the stochastic approximation method based on the observed productivity and species richness at a regional scale in boreal and temperate forests spanning different degrees of latitude, along an elevation gradient (500–4000m) in Ecuador [21], in the Guadalquivir River delta in Spain, and in natural temperate forests in the Czech Republic, Poland, and Slovakia spanning different degrees of longitude [17](Text C in S1 File).

### Derivation and verification of the forms of the SRPR

#### Local scale

The local scale is the spatial extent and grain within a local community [59,60]. The local community is defined as a set of species that occupy a single relatively homogeneous habitat within a landscape [59,60]. There is often a variety of habitat types within the landscape, such as valleys, hills, croplands, coastal belts, or wetlands [59,60]. When plots or quadrats are used to test the SRPR in a local community, the levels of resource availability (*R*_a_) in these plots can be seen as relatively identical, i.e., *R*_a_ = 0 in Eqs 1–11. Therefore, the primary productivity, *P*(s), is dictated by *u*(s) and *m*(s) and *D* in Eq 1. I substituted the values of the process parameters (i.e., the first value in each cell of the data columns with # in Table 1, which were estimated by the stochastic approximation method)into Eq 11 (the solution of Eq 1) to derive the different forms of SRPR at the local scale.

To verify the derived forms of the SRPR, I used the observed data of the five typical SRPR forms in local communities (Text B in S1 File) to estimate the values of the process parameters(the second value in each cell of the data columns with # in Table 1)by both the least-square method and the stochastic approximation method [61]. Then, I substituted plant species richness and the values of these estimated process parameters into Eq 11 to derive the SRPR forms, and compared them with the five typical SRPR forms using at-test and goodness-of-fit test. The substituted plant species richness showed a continual gradient, which was the same as that in the five typical SRPR forms. The five typical forms included the asymptotic, positive, humped, negative and irregular forms of the SRPR that were respectively observed in experimental grasslands of Europe [62], the floodplain of the river Saale near Jena in Germany[63], the grasslands in Texas [64], and natural plant communities dominated by vascular plants in Gloucestershire of the UK [15], and the Czech Republic [22], respectively (Text B in S1 File). The dynamics of SC effect, *u*(s), density effect, *m*(s), and the interspecific competition stress, *N*, in these observed SRPR forms were presented based on Eqs 5, 6 and 7.

#### Regional scale

The regional scale is the spatial extent and grain across different local communities within a metacommunity or across different biogeographical provinces [59]. The metacommunity is a set of local communities linked by dispersal of multiple interacting plant species within a landscape or a vast region [59,60]. The strengths of the processes affecting the SRPR often change with the spatial extent and grain, and correspondingly the SRPR also changes with the scales [20]. Thus, I further tested the SRPR forms across different local communities distributed in different biogeographical provinces. It is clear that the levels of resource availability (*R*_a_) are different among local communities in different biogeographical provinces, i.e., *R*_a_≠0 in Eqs 1–11[20,59]. I assumed that there was a long transect across six zonal forests, including deciduous coniferous forests (DCF), evergreen needle-leaf forests (ENF), deciduous broad-leaved forests (DBF), evergreen coniferous and broad-leaved mixed forest (ECB), evergreen broad-leaved forests (EBF), and tropical rain forests (TRF) from as far north as Russia to as far south as China[66,67]. The levels of resource availability (*R*_a_) are greatly variable among these forests. Specifically, lower heat resources and precipitation are generally observed at higher latitudes compared with lower latitudes [67,68]. Consequently, mineral nutrients in soil are relatively richer in southern forests than northern forests, which lead to a *R*_a_ gradient from circumpolar latitudes to the equator [67,68]. Moreover, plant species richness in these forests also increases from north to south [66,67]. Under these conditions, I assumed that there were the five SRPR forms in local communities within the sezonal forests because of different strengths in the *u*(s), *m*(s), and *N*at local scales. I further derived the different forms of the SRPR in the local communities within these forests with the addition of an increment to *R*_a_ in Eq 11 in the southern forests compared with the northern forests. However, other values of the process parameters were the same as Table 1 in derivation. Lastly, the same derived forms of the SRPR occurring in all these forests were superposed along a species richness gradient from north to south (the assumed long transect) and indicated the changes of the SRPR with sampling methods by a graphical method.

To verify the derived forms of the SRPR, I used the observed data of the five typical SRPR forms (Text C in S1 File) in four regional studies to estimate the values of the process parameters (the third value in each cell of the data columns with # in Table 1) by both the least-square method and the stochastic approximation method [61]. Then, I substituted plant species richness and the values of these estimated process parameters into Eq 11 to derive the SRPR forms, and compared them with the five observed SRPR forms at regional scales using at-test and goodness-of-fit test. The substituted species richness showed a continual gradient, which was the same as that in the five observed SRPR forms (Text C in S1 File). The dominant asymptotic form was verified using data from the boreal and temperate forests of the Swedish National Forest Inventory and the Swedish Survey of Forest Soils and Vegetation, which included approximately 4,500 permanent tracts, covered an area of 400,000 km^2^ and spanned13.7 degrees of latitude[69]. The SRPR data collected from Ecuador was used to test the positive and humped forms [21], and included 6,175 fern individuals from 91 species and 560 trees, in 18 plots along an elevation gradient (500–4000 m). Verification of the irregular SRPR form was conducted with the data from over 100 permanent plots located in natural temperate forests in the Czech Republic, Poland, and Slovakia [17]. The negative SRPR form was verified using the relatively old data of biomass production and species richness from the Guadalquivir River delta (SW of Spain), formed by fluvio-marine sediments filling up the estuary during the Holocene[70]. Please refer to Text C in S1 File for more details regarding the specifics of these applications. The dynamics of SC effect, *u*(s), density effect, *m*(s), and the interspecific competition stress, *N*, in these observed SRPR forms were presented based on Eqs 5, 6 and 7.

## Results

### The forms of the SRPR at a local scale

**Fig 1:**
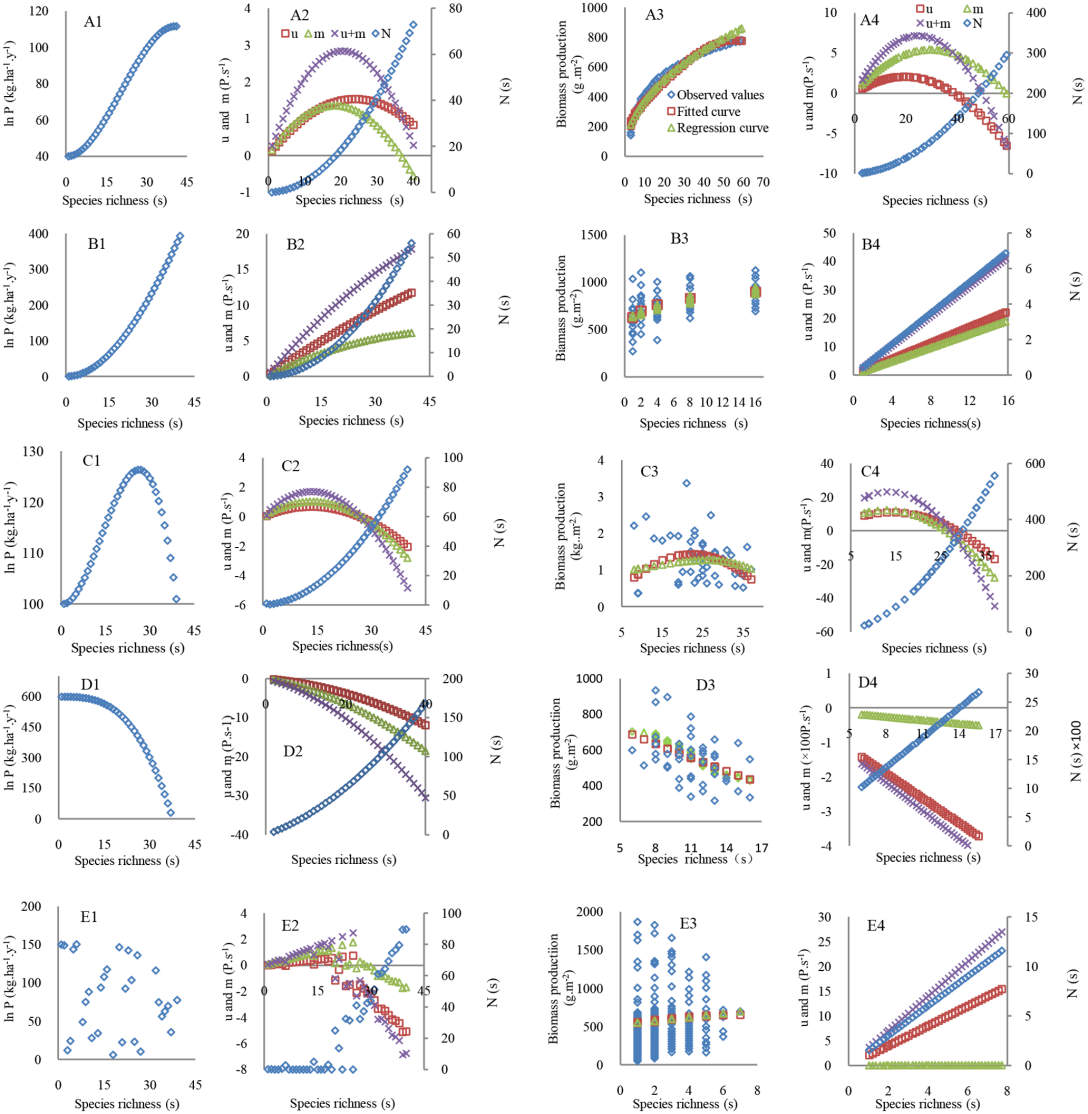
The asymptotic, positive, humped, negative and irregular SRPRs at a local scale controlled by different strengths of SC effect, *u*(s), density effect, *m*(s), and the interspecific competition stress *N*. A1, B1, C1, D1, and E1 were drawn based on the predicted productivity by the first value of the process parameters in each cell of the data columns with #in Table 1 being substituted into Eq 11. A2, B2, C2, D2, and E2 are the dynamics of *u*(s), *m*(s), and *N* corresponding to these different SRPR forms: A1, B1, C1, D1, and E1. A3, B3, C3, D3, and E3 are the observed values of primary productivity along the species richness gradients at local sites in experimental grasslands of Europe [62], the floodplain of the river Saale near Jena in Germany [63], the grasslands in Texas [64], natural plant communities dominated by vascular plants in Gloucestershire of the UK [15], and the Czech Republic [22], respectively (Text B in S1 File). Regression curves are fitted based on the observed primary productivity and fitted curves are drawn using the predicted primary productivity by the second value in each cell of the columns with # in Table 1 being substituted into Eq 11. A4, B4, C4, D4, and E4 indicate the dynamics of *u*(s), *m*(s), and *N* in the five studies described above.

When the ecological processes were at different strength levels (i.e. the first value in each cell in the data columns with # in Table 1), the five forms of the SRPR were derived from Eq 11 (Fig 1). With increasing species richness as shown on the x-axis in Fig 1, the different SC effect, *u*(s), density effect, *m*(s), and competition stress, *N*, on primary productivity were also given. (I) Asymptotic form (Fig 1A1), which occurred when both *u*(s) and *m*(s) or their sum was greater than 0 (Fig 1A2) and the strengths of *u*(s) and *m*(s) on primary productivity were greater than that of *N* based on Eqs 2 and 3. In this form, *dP*/*ds* in Eq 1 was greater than 0 and ln *P* presented an increasing trend. However, *N* was continually strengthened with increasing *s*in Eqs 4 and 5, and consequently the effects of *u*(s) and *m*(s) were weakened. When *u*(s) + *m*(s) crossed the x-axis, i.e., was equal to 0 (Fig 1A2), *dP*/*ds* was equal to 0 and the ln *P* increased to its greatest value.

(II) Positive form (Fig 1B1)occurred when *u*(s) and *m*(s) or their sum was much greater than 0 (Fig 1B2) and their effects on the ln *P* always increased from low to high species richness levels. *dP*/*ds* was always greater than 0 in Eq 1, and ln*P* presented a nearly linear increase, although *N* obviously increased, which decreased *u*(s) + *m*(s) at high species richness.

(III) Humped form(Fig 1C1) occurred when *u*(s) and *m*(s) first increased and then decreased, and their sum equaled zero at approximately a species level of 27(Fig 1C2). The sum of *u*(s) and *m*(s)subsequently became negative, which made *dP/ds* positive, zero and negative in Eq 1, and ln *P* first reached a peak and then decreased. When *u*(s) + *m*(s) were negative, ln*P* rapidly declined because of the steeply increasing *N*.

(IV) Negative form (Fig 1D1), which occurred when the high monoculture productivity, *P*_m_, resulted in a large *N* and led to negative *u*(s) and *m*(s) (Fig 1D2). *dP*/*ds* in Eq 1 was always less than or equal to 0 (Eq 1) and ln *P* did not present an increase at any species richness. At high species richness, *N* then became greater because of intense interspecific competition, thus leading to a rapid decline in ln*P*.

(V) Irregular form(Fig 1E1) occurred when there were disturbances with different intensities. In these circumstances, *u*(s), *m*(s) and *N* were less than, greater than, or equal to0, and these changes were irregular (Fig 1E2). Consequently, ln *P* was also irregular with increasing *s* (Fig 1E1). In Fig 1, if A1 and B1 were considered as the same types of SRPR based on high similarity, then there were two positive SRPR relationships, indicating a generally positive pattern. In Fig 1C1, if a section of the species richness gradient was considered, such as 20 species that commonly occurred in a natural ecosystem in an era of high species extinction, then a positive SRPR was indicated. Thus, a positive SRPR was dominant and easily observable.

Statistical tests indicated that, for the asymptotic form, there was no a significant difference between the observed productivity in experimental grasslands of Europe and the fitted productivity (*t* = 1.75, *df* = 49, *p* = 0.81; *X*^2^ = 37.79, *df* = 49, *p*>0.90, Fig 1A3; Text B in S1 File). These tests supported the theoretical derivation of the forms of the SRPR. The magnitudes of the estimated values of the process parameters representing the strengths of the ecological processes in Table 1 (the second value in each cell of the asymptotic form column with #) partially explained the observed asymptotic form of the SRPR in the experimental grasslands. There were also no significant differences between the observed and fitted productivity for the positive form in Germany (*t* = 0.63, *df* = 57, *p* = 0.52;*X*^2^ = 61.74, *df* = 57, *p*>0.25; Fig 1B3), the humped form in the grasslands of Texas (*t* = 1.16, *df* = 18, *p* = 0.20; *X*^2^ = 13.40, *df* = 18, *p*>0.50; Fig 1C3), the negative form in Gloucestershire of the UK(*t* = 0.76, *df* = 44, *p* = 0.44; *X*^2^ = 57.52, *df* = 17, *p*>0.05; Fig 1D3) and the irregular form in the Czech Republic(*t* = 0.35, *df* = 164, *p* = 0.73; *X*^2^ = 20.28, *df* = 164, *p*>0.995; Fig 1E3). To some degree, these estimated values of the process parameters, corresponding to the different forms of the SRPR in Table 1, also explained the strength of the ecological process affecting the observed SRPR at local sites.

The dynamic characteristics of SC effect, *u*(s), density effect, *m*(s), and the interspecific competitive stress, *N*(s), affecting the observed asymptotic, positive, humped and negative forms of the SRPR at local scales (Fig 1A4, 1B4, 1C4 and 1D4) were highly similar to those on the five derived typical forms of the SRPR (Fig 1A2, 1B2, 1C2 and 1D2), except for the irregular form (Fig 1E2 and 1E4). For all five observed forms of the SRPR, the interspecific competitive stress, *N*, presented a continual increase with increasing *s*(Fig 1A4, 1B4, 1C4, 1D4 and 1E4). However, SC effect and density effect and their sum on the observed asymptotic and humped forms of the SRPR (Fig 1A4 and 1C4) firstly increased and then decreased with increasing *s*. SC effect and density effect and their sum on the positive and irregular forms of the SRPR (Fig 1B4 and 1E4) presented a continual increase with increasing *s*. Conversely, these effects on the observed negative form of the SRPR presented a continual decrease, indicating that the dominant negative effect controlled the form of the SRPR (Fig 1D4).

### The forms of the SRPR at a regional scale

**Fig 2:**
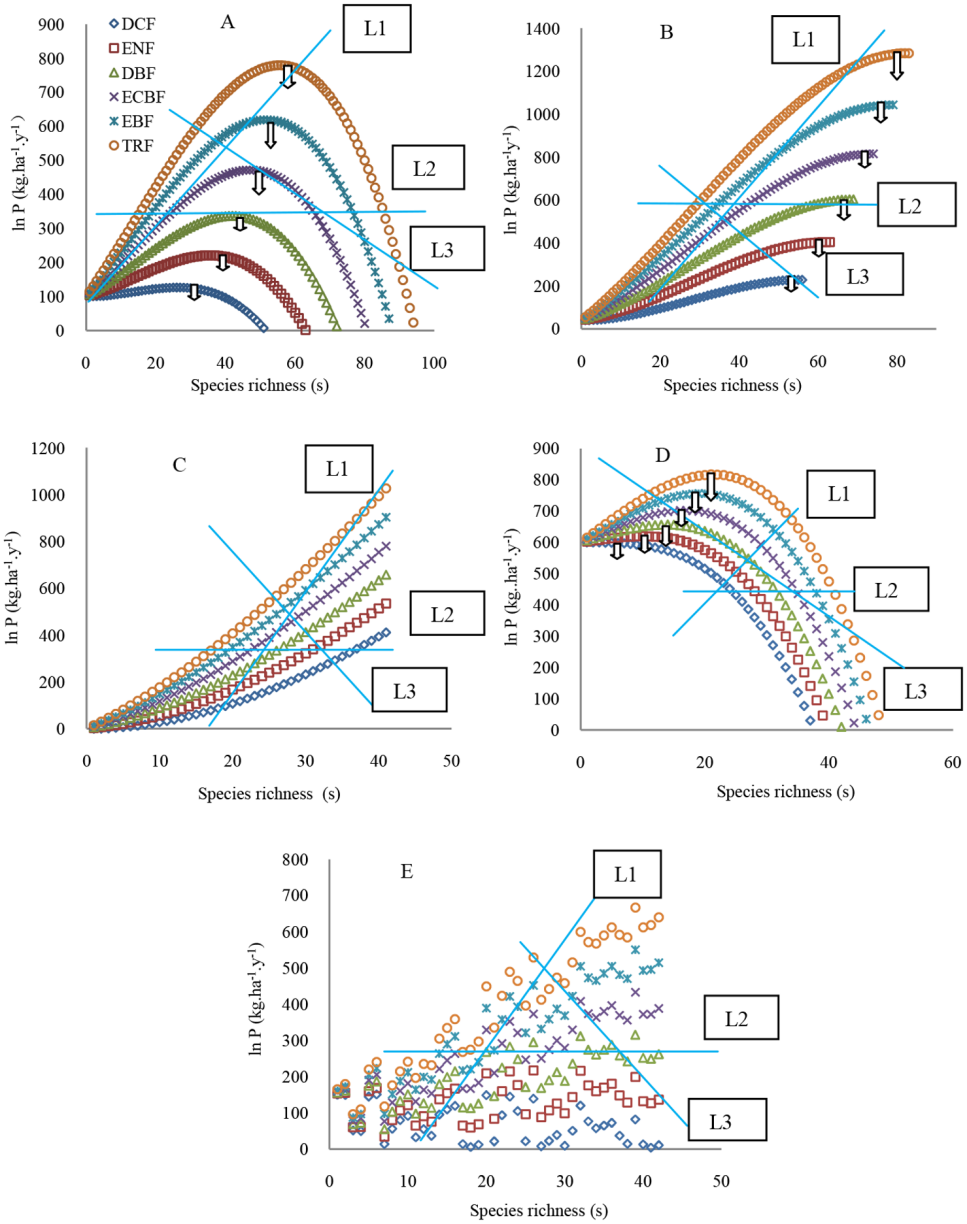
The derived forms of the SRPR across the local communities of the zonal forests in different biogeographic provinces from north in Russia to south in China. A: Humped; B: Asymptotic; C: Positive; D: Negative; E: Irregular forms. L1, L2, and L3 are three types of sampling methods. DCF: deciduous coniferous forests; ENF: evergreen needle-leaf forests; DBF: deciduous broad-leaved forests; ECBF: evergreen coniferous and broad-leaved mixed forest; EBF: evergreen broad-leaved forests; and TRF: tropical rain forests [68]. A-D were drawn based on the calculation results that the first value of the process parameters in each cell of the data column# in Table 1 were substituted into Eq 11 but the parameters *R*_a_ and *μ* did not equal zero. The *R*_a_ was respectively assigned 0, 30, 60, 90, 120, and 150 for the forests DCF, ENF, DBF, ECBF, EBF, and TRF. *μ* equaled 0.1. Each curve in Fig2A, B, C, D, or E represents the similar SRPR forms occurring in the local communities of these zonal forests. In D, there is a rising section of ln*P* at low species richness except for the forests DCF and ENF. The species richness on the x-axis directed by arrows hanging on the curves is the greatest species richness; however, C does not display the greatest richness.

The derived forms of the SRPR occurring in the local communities in DCF, ENF, DBF, ECBF, EBF, and TRF from north to south still included the five typical types (Fig 2A–2E). This derivation verified the assumption in the Methods, when the *R*_a_ in Eq 11 had been assigned greater values in the southern forests than northern forests. However, the same forms of the SRPR were significantly different among different forests in the maximum values of the primary productivity and species richness (each of the curves in Fig 2A–2E). The irregular forms of the SRPR changed into the positive forms in ECBF, EBF and TRF (Fig 2E) due to the significantly increasing resource availability *R*_a_. Comparatively, the irregular forms of the SRPR did not show a great change in the DCF, ENF and DBF with relatively small increases of *R*_a_ (Fig 2E).

Furthermore, when quadrats were assumed to be placed along the transect as the line L1 across these zonal forests(Fig 2A–2E), in which low and high richness respectively corresponded to low and high productivity levels, then a positive SRPR form across these forests might be observed. When quadrats were placed as the line L2 across these zonal forests(Fig 2A–2E), in which low and high richness corresponded to similar productivity levels, then an irregular SRPR form might be observed. When quadrats were placed as the line L3 across these zonal forests, in which low richness corresponded to high productivity and high richness corresponded to low productivity, then a negative form might be observed. It was noted that when quadrats were set from low to high species richness along the line L1, andthen along the line L2 at the intersection of the two lines (Fig 2A–2E), an asymptotic SRPR was observed. When the quadrats were first set along L1 and then L3, a humped SRPR was observed. Therefore, there were the various SRPR forms across different biogeographical provinces.

**Fig 3:**
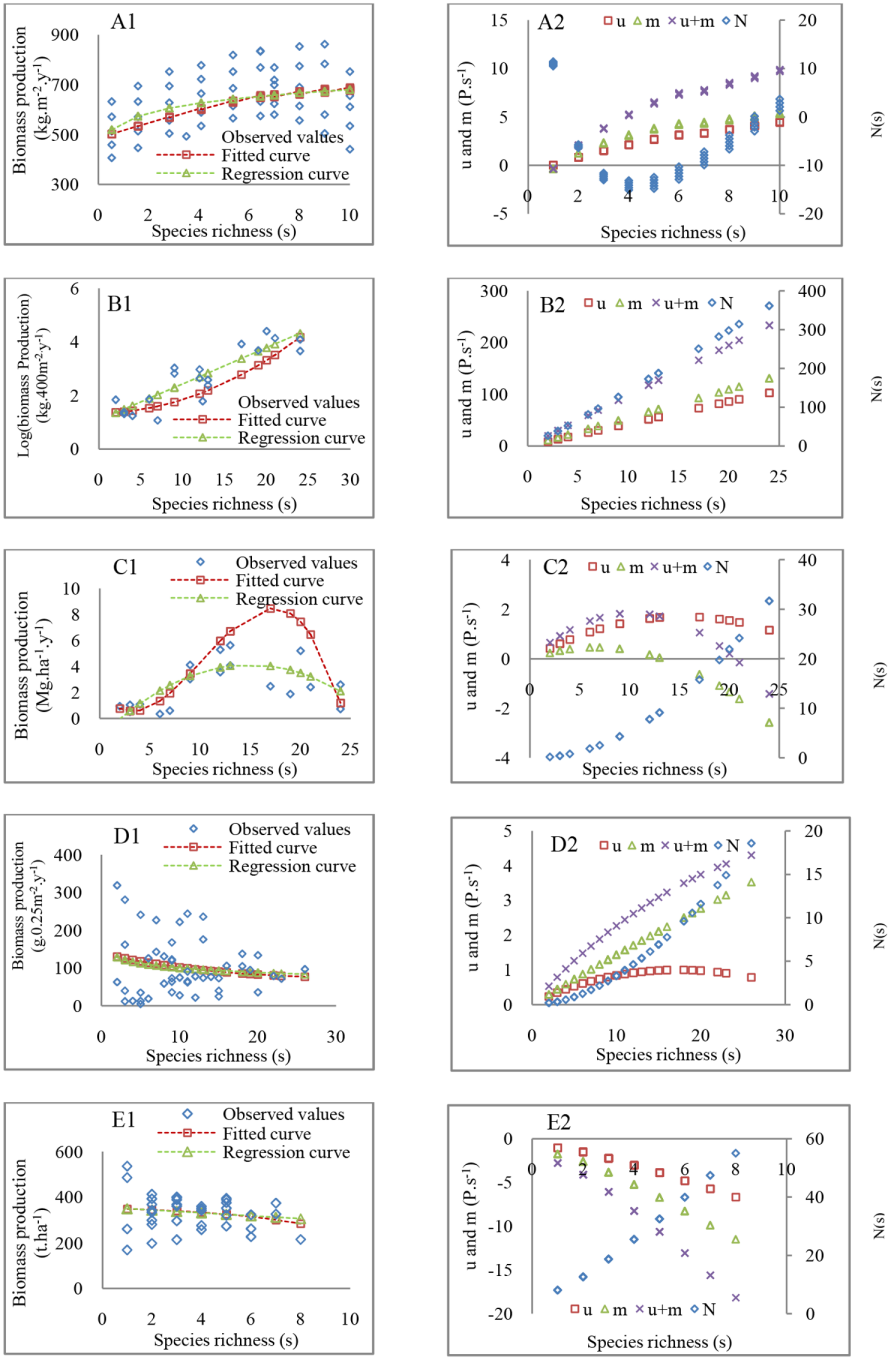
The verification of the derived forms of the SRPR at a regional scale and the dynamics of SC effect, *u*(s), density effect, *m*(s), and competition stress, *N*(s). A1, B1, C1, D1, and E1 represent the asymptotic, positive, humped, negative, and irregular forms of the SRPR, respectively. Observed values are the levels of primary production actually observed along a species richness gradients, successively including boreal and temperate forests (asymptotic) in the Sweden [66], trees (humped) and ferns (positive) in 18 study plots along an elevation gradient (500–4000 m) in Ecuador [21], herbs (negative) in Guadalquivir River delta in Spain [67], and woody plants (irregular) in over 100 permanent plots in natural temperate forests in the Czech Republic, Poland and Slovakia [17] (Text C in S1 File). The fitted curves are the derived results produced by substituting the third value in each cell of the data columns with # in Table 1 into Eq 11. The regression curves are the results predicted by regression of these observed levels of primary production on species richness.

In verification of the derived forms of the SRPR across different regions, there were no significant differences between the predicted and observed productivity along a same species richness gradient for the dominant asymptotic forms (*t* = 1.5, *df* = 49, *p* = 0.12; *X*^2^ = 41.24, *df* = 49, *p*>0.5,Fig 3A1; Text C in S1 File). For the positive form of the SRPR, there were no significant differences between the predicted and observed productivity along a same species richness gradient within a metacommunity (*t* = 1.32, *df* = 18, *p* = 0.18, Fig 3B1), but goodness-of-fit test showed significant differences (*X*^2^>35, *df* = 18, *p*<0.01). Both the t-test and the goodness-of-fit test indicated no significant differences between the predicted and observed productivity in the humped, negative and irregular forms along different species richness gradients (*t* = 1.61, *df* = 18, *p* = 0.11; *X*^2^ = 15.26, *df* = 18, *p*>0.50, Fig 3C1; *t* = 0.19, *df* = 48, *p* = 0.8; *X*^2^ = 7.03, *df* = 48, *p*>0.995, Fig 3D1; *t* = 0.26, df = 48, *p* = 0.93; *X*^2^ = 2.91, *df* = 36, *p*>0.995, Fig 3E1). These results indicated that the observed SRPR forms at regional scales could be well fitted by Eq 11. The estimated values of the process parameters representing the strengths of the ecological processes (the third value in each cell of the data columns with #) also, to some degree, explained the five observed SRPR forms. The estimated *a* and *b*, the strengths of SC effect, *u*(s), and density effect, *m*(s), which were used to derive the asymptotic and positive forms, were obviously greater than those to derive the other SRPR forms (Table 1).

In the dynamic characteristics, the interspecific competitive stress, *N*(s), affecting the observed positive, humped, negative, and irregular forms of the SRPR at a regional scale (Fig 3B2–3E2) presented continual increases with increasing species richness. However, the *N*(s) affecting the observed asymptotic form of the SRPR first presented a decrease and then an increase, a bit different from other forms (Fig 3A2). The dynamic SC effect, *u*(s), and density effect, *m*(s), and their sum affecting the observed asymptotic, positive and negative forms of the SRPR indicated continual increases with the increasing species richness (Fig 3A2, 3B2 and 3D2), but for the irregular form of the SRPR, *u*(s) and *m*(s) indicated a continual decrease (Fig 3E2). The change of *u*(s), *m*(s), and their sum affecting the humped form of the SRPR first presented an increase, then a decrease (Fig 3C2). The dynamics of *u*(s), *m*(s), and *N*(s)affecting the negative and irregular forms of the SRPR at the regional scale were different from those at the local scale (Figs 3D2, 3E2, 1D4 and 1E4).

## Discussion

With a mathematical combination, I have demonstrated how the forms of SRPR change with strengths of the ecological processes affecting species richness and primary productivity [5,6,10–12]. The combination includes assessments of the positive and/or negative effects of these ecological processes on primary productivity and species richness, and establishment of the combination models containing these processes. I further derive the forms of the SRPR when parameters are assigned different values within these combined processes and then verify these forms of the SRPR using observed data from third parties. I also present the dynamics of three comprehensive processes (i.e., SC effects, density effects and interspecific competition stress) structured by other processes. The derived and verified forms of the SRPR change with the strengths of the combined processes, indicating that the strengths of ecological processes determine the forms of the SRPR.

Three comprehensive processes are as follows: (1) SC effect, *u*(s) (Eqs 2 and 6), which occurs when species richness increases in a plant community with low species richness levels, there may be no or little competition stress (ln *N*, Eq 2). *u*(s)is primarily dependent on the term *as*, i.e., a monotonous increase with increasing species richness, which yields increasing primary productivity. However, when species richness increases to higher levels, more competitors occur and the competition stress, ln *N*, weakens the term *as*(Eq 2). Consequently, this makes *u*(s) decrease or even become negative, and causes decreases in primary productivity. (2) Density effect, *m*(s) (Eqs 3 and 7), also changes with SC effects. When the individual numbers and productivity of plants are very small, there is no or little competition stress, ln *N* (Eq 3) [9,35–38]. Consequently, *m*(s) approximately equals *bs*, and shows a positive linear effect on productivity with increasing species richness, *s*. However, when the individual numbers and productivity of plants become greater over time and space, the interspecific competition stress, *N*, that may result in decreased primary productivity must be considered. In such circumstances, density effect is weakened or even becomes negative (Eq 3). (3) Interspecific competition stress, *N*, (Eqs 4 and 5), always presents a positive increase with increasing species richness and productivity. The *N* weakens *m*(s) and *u*(s) on the primary productivity with species richness and productivity (Eqs 2 and 3). Thus, *N* results in decreased primary productivity. Many control experiments and field investigations indicate that the SRPR are primarily positive and asymptotic forms [3,10,70,71]. Based on the combination model, the two forms occur because the interspecific competition stress does not play a significant role in the regulation of *m*(s) and *u*(s) on primary productivity. In fact, competition stress as negative feedback is particularly important in a natural world [38,56,58].

Clearly, changes in the three comprehensive processes are inherent with increases or decreases of species richness and primary productivity based on Eqs 2, 3, 6 and 7. Variation in species richness and primary productivity may be generated due to stochastic and deterministic processes, for example, by a selection of plots at different successive stages in a local community or by artificially manipulated experiments of plant species[2,72]. As a result, different types of SRPR (Fig 1)canbe derived using Eqs 11 when SC effect, *u*(s), density effect, *m*(s), and the interspecific competition stress, *N*, arise at the levels of the different strengths through assigning different values to the process parameters (Table 1). (1) The positive form is based on greater *as* and *bs*, i.e., positive SC effects and density effects (Eqs 3 and 4) are far stronger than *N*. Consequently, the SRPR is controlled by the positive *u*(s)and *m*(s) along the species richness gradient. (2) The asymptotic form is similar to the positive form with the positive SC (*as*) and density effects (*bs*) being stronger than *N* at a low species-richness level. However, the *as* and *bs* are offset by *N*, at a high species-richness level. (3) The humped form is based on the positive sections (*as* and *bs*) of SC effect and density effect being far greater than *N* (Eqs 2 and 3) at a low species-richness level, leading to the increasing section of the SRPR. Conversely, with increasing species richness, *N* exceeds *as* and *bs*, resulting in the decreasing section of the SRPR and creating the humped pattern. (4)The negative form is that in which there is high productivity at low species richness levels, and consequently the interspecific competition stress, *N*, is much greater than the sum of *as* and *bs*, which generate the negative *u*(s) and *m*(s) (Eqs 2 and 3), and the negative SRPR. (5) The irregular form is generated through disturbance, which is a key factor regulating almost all processes, which makes the SRPR change irregularly. These results indicate that the form of the SRPR is diverse rather than a single pattern, and the process strengths can explain why different types of the SRPR have existed in the vast amount of data from field investigations and artificially manipulated experiments of plant species throughout the past decades [1,27,40,70,73–75].

Verification of the SRPR forms at a local scale also supports the points that the process strengths determine the forms of the SRPR. Specifically, the data of the five typical observed studies of the SRPR (Text B in S1 File) are used to estimate the parameter values of the ecological processes in the combination models (Table 1). The five observed SRPR forms can be well fitted by the five derived forms of the SRPR (Fig 1A3–1E3), which indicate that Eq 11 may be applied to the prediction of the five typical forms of the SRPR. More importantly, the relative sizes of the estimated values of the process parameters (Table 1) can to some degree represent the effect strengths of ecological processes. The dynamics of the three comprehensive processes discussed above can explain the different observed forms of the SRPR. In the last century, different forms of the SRPR have been compiled from numerous data sources in which the asymptotic and positive forms were dominant and the irregular form arose in about 21% of all studies [1–3, 10]. Since the beginning of the 21^st^ century, the observed frequency of the humped [12,13,18,19,21], negative [15,17,22] and irregular [14,16,20,23] forms of the SRPR in studies have obviously increased, as have the dominant asymptotic and positive forms [63,72]. These field studies support the theoretical derivation of the SRPR. The data from these studies can be used to quantify the process strengths occurring at respective study sites based on Eq 11 to elucidate the mechanisms underlying the different forms of the SRPR.

Furthermore, I assume that all SRPR forms occur in local communities within the typical forests distributed in different climatic zones due to effects of the different strengths of ecological processes on the SRPR at the local scales. Then, the SRPR forms at regional scales are derived and verified. On the regional scale level, the variation of resource availability, *R*_a_, is significant. The derived forms of the SRPR also include various types of the forms with different *R*_a_, when the sampling is assumed to be conducted across typical forests. The further verification of the derived forms of the SRPR is also based on the study data at regional scales, which include boreal and temperate forests spanning different degrees of latitude [66], along an elevation gradient (500–4000m) [21], in Guadalquivir River delta in Spain [67], and in natural temperate forests in the Czech Republic, Poland and Slovakia spanning different degrees of longitude [17](Text C in S1 File). Results indicate no significant differences between the derived and observed forms of the SRPR (Fig 3). Parameter estimation also reflects the variation of strengths of processes affecting the respective observed forms of the SRPR. Because of differences in estimated values of process parameters, the dynamics of SC effect, *u*(s), density effect, *m*(s), and the interspecific competition stress, *N*(s), show diverse characteristics (Fig 3A2–3E2). It is noted that the data collected from the tree layers and fern layers in a set of plots along an elevation gradient in Ecuador were used to verify the observed positive and humped forms of the SRPR (Fig 3B1 and 3C1) [21] (Text C in S1 File). The dynamics of *u*(s), *m*(s) and *N* (s), which affect the positive and humped forms observed (Fig 3B2 and 3C2), result in obviously different forms due to the different strengths of the ecological process (Table 1) in the forest canopies and fern communities under the canopies, two contrasting mesohabitats. These results further support process strengths determining the forms of the SRPR. Moreover, there are obvious responses in strengths of the ecological processes (Table 1) to different scales. Consequently, the forms of the SRPR at a local scale (Fig 2) influenced by these processes can change into other forms (Figs 2 and 3) with an expanded scale, indicating the scale dependence.

Loss of plant diversity has undoubtedly impaired ecosystem function. However, there is a long-term debate on the SRPR sustaining the ecosystem functions, due to inconsistent results in the SRPR observations and a variety of ecological processes or mechanisms to explain the SRPR. The implications of the study are that, to resolve the controversy and further provide sound predictions of how the SRPR responds to ecological processes, it is essential to combine effects of key ecological processes on the SRPR. The forms of the SRPR on different scales can be quantified by considering changes in strengths of the ecological processes regulating species richness and primary productivity in combination models. In the future, it would be worthy to consider modeling the temporal dynamics of species richness, plant productivity, all processes affecting SRPR and their interactions, such as those in succession. After all, species richness, plant productivity, and all processes affecting SRPR are changing with time. This can help clarify the SRPR more than changes of the SRPR forms at local and regional scales in the study, as suggested by a reviewer. Additionally, although the study indicates some negative or insignificant effects of plant species richness at a high species richness level on primary productivity, plant diversity has numerous positive effects on ecosystem functioning.

## Supporting information

S1 File The file contains Texts A, B and C. Text A presents the methods of t-test and the goodness-of-fit test for assessing the differences between the derived and observed productivity along plant species richness gradients. Texts B and C, respectively, indicate the sources and description of the observed data that were used to verify the five derived plant species richness-productivity relationship (SRPR) forms at the local and regional scales.
(DOCX)
