# Correction: Process strengths determine the forms of the relationship between plant species richness and primary productivity

**DOI:** 10.1371/journal.pone.0208478

**Published:** 2018-11-29

**Authors:** Zhenhong Wang

In the Materials and Methods section, there is an error in the first equation under the section titled “Combination model.” Please view the complete, correct equation here:

$$\frac{dP}{ds}=u(s)P+m(s)P‑\tau DP+\mu R_{a}P$$
