# Supplementary material for: Process strengths determine the forms of the relationship between plant species richness and primary productivity
Source: PLoS One. 2017 Nov 15;12(11):e0185884. doi: 10.1371/journal.pone.0185884 (PMC5687741; doi:10.1371/journal.pone.0185884)
Supplement: S1 File — Text A presents the methods of t-test and the goodness-of-fit test for assessing the differences between the derived and observed productivity along plant species richness gradients. Texts B and C, respectively, indicate the sources and description of the observed data that were used to verify the five derived plant species richness-productivity relationship (SRPR) forms at the local and regional scales. (DOCX) [file pone.0185884.s001.docx]

Dear PLOS ONE staff,

      Thank you for your email. Thank you for your conscientiousness for my manuscript as well.

Per your requests, I solved the following problems: 1. one of the hybrid problems in equations;   2. The first citation cannot occur in the footnotes. However, the lost spaces between words that had been recovered in FTC2 last time occured again in the version you sent. I remain no change.

      Additionally, in last update, four  surplus symbols occur on 11 page. Specifically, in the equation 8, the equation code "(8)"  needs to be deleted; in equation 9, "(" also needs to be deleted; in the last two lines of Page 11, “ln” and "C" needs to be deleted to solve another hybrid problem in equation.    But the document I have revised cannot be save and upload by system. So, I upload a compress file containing PONE-D-16-28570R2_FTC3 and Supporting Information to you by the email. Please kindly help me update. Or unlock the document, let me to upload.

      Have a nice day!

      Zhenhonhg

The following section includes four symbols to be deleted on the 11 page:

$\frac{dP}{ds}=[(a+b)s-(k1+k2) (0.5 b_{1}s^{2}+b_{2}P_{m}s-\rho D\ln\left( s \right)) ]P-\tau DP + \mu R_{a}P$ (8)

The variables in equation 8 can be separated and *P*(s) can be integrated as follows:

$\ln P=C+ \frac{1}{2}\left( a+b \right)s^{2}-\left( k_{1}+k_{2} \right)\left[ \frac{1}{6}b_{1}s^{3}+{\frac{1}{2}b}_{2}P_{m}s^{2}-\rho D\left( s\ln\left( s \right)-s \right) \right]-\tau Ds+\mu R_{a}s$ (9)

where *C* is an integration constant. When *s* = 0, ln*P* = *P*_0_ =*P*_m_=0; then, *C* = 0, and equation 9 changes as follows:

$\ln P=\frac{1}{2}\left( a+b \right)s^{2}-\left( k_{1}+k_{2} \right)[\frac{1}{6}b_{1}s^{3}+{\frac{1}{2}b}_{2}P_{m}s^{2}-\rho D(s\ln\left( s \right)-s)]-\tau Ds+\mu R_{a}$ (10)

When *s* = 1, ln *P* = *P*_1_ = *P*_m_, then *C* = *P*_m_-$\frac{1}{2}\left( a+b \right)+\left( k_{1}+k_{2} \right)$($\frac{1}{6}b_{1}$+$\frac{1}{2}{b_{2}P}_{m}$+ 1) + *τD*-$\mu R_{a}$*,* equation 9 changes as follows:
